# A New Threat Looming over the Mediterranean Basin: Emergence of Viral Diseases Transmitted by *Aedes albopictus* Mosquitoes

**DOI:** 10.1371/journal.pntd.0001836

**Published:** 2012-09-27

**Authors:** Giuliano Gasperi, Romeo Bellini, Anna R. Malacrida, Andrea Crisanti, Michele Dottori, Serap Aksoy

**Affiliations:** 1 Dipartimento di Biologia e Biotecnologie, Università di Pavia, Pavia, Italy; 2 Medical and Veterinary Entomology, Centro Agricoltura Ambiente “G. Nicoli”, Crevalcore, Bologna, Italy; 3 Division of Molecular and Cell Biology, Imperial College, London, United Kingdom; 4 Dipartimento di Medicina Sperimentale, Università di Perugia, Italy; 5 Istituto Zooprofilattico Sperimentale della Lombardia e dell'Emilia-Romagna, Brescia, Italy; 6 Yale School of Public Health, Department of Epidemiology and Public Health, New Haven, Connecticut, United States of America

Until the 1960s, many areas of southern Europe were still plagued with malaria, a major vector-borne disease transmitted by mosquitoes of the genus *Anopheles*, including *An. labranchiae*, *An. sacharovi* (*maculipennis* complex), and *An. superpictus* [1], [2]. Of all diseases, malaria has made the strongest claim for attention as the leading problem of public health throughout the modern era. As an endemic and epidemic disease, malaria held nearly the whole of the Italian peninsula as well as the islands of Sicily and Sardinia under its sway [1]. For almost a century, malaria was capable of erupting with epidemic fury every season and caused death and widespread disease. Malaria became an integral factor at all levels of the Italian society—in fact, the word “malaria” comes from the medieval Italian, meaning “bad air” [1]. Malaria had become a classical social and occupational disease, and a disease of poverty and societal neglect. The southern Italian politician Francesco Saverio Nitti (1868–1953) emphatically argued that “malaria lies at the root of the most important demographic and economic facts”, and that “its contest (contest of malaria) was the necessary precondition for resolving all other problems affecting the region” [1]. Elimination of malaria from the European continent and in particular from Italy, the most affected region, has been the result of more than 50 years of efforts directed at reducing vector populations. This translated into an unprecedented environmental management to reduce mosquito habitats and culminated with the introduction of insecticides after the end of World War II. The last indigenous case of malaria in Italy was recorded in Sicily in 1962 [3].

Now in 2012, a new threat for vector-borne diseases has emerged on the horizon for southern Europe. The Asian tiger mosquito, *Aedes albopictus*, is currently the most invasive mosquito species in the world. Over the past 30 years, this aggressive day-biting mosquito has rapidly spread from its native tropical forests of Southeast Asia across the world and is found currently in at least 28 countries in all continents, except Australia and Antarctica [4], [5]. Its populations exhibit extreme variation in adaptive traits such as egg diapause, cold hardiness, and autogeny (the ability to mature a batch of eggs without blood feedings) [6]. This high ecological plasticity permits this species to spread and successfully establish in both tropical and temperate regions. The colonization of Europe by *Ae. albopictus* began in Albania in the late 1970s [7], then in Italy in the 1990s [8], and gradually spread into the other Mediterranean countries, including France, Spain, Slovenia, Croatia, Bosnia and Herzegovina, Montenegro, and Greece [5]. Tiger mosquitoes have also established permanently in southern Switzerland, and hence there is considerable concern about possible outbreaks further north [9]. The current distribution map of *Ae. albopictus* in Europe can be seen on the European Centre for Disease Prevention and Control (ECDC) website [10]. Predictive models indicate its likely expansion throughout Europe due to climate change [11]–[13].

The dramatic global expansion of this aggressive mosquito has increased public health concern due to its ability to transmit numerous arboviruses, including the most prevalent arboviral pathogens of humans: chikungunya and to a lesser extent dengue viruses [14]–[17]. After the 2002 epidemic in the United States of America [18], it is thought that *Ae. albopictus* may also play a role as an important bridge vector of West Nile virus, a pathogen that has become endemic in northeastern Italy [19]–[21]. The frequent introductions of dengue and chikungunya virus in Europe by infected travellers further indicate an increase in the risk of arbovirus outbreaks in Western regions [22]–[25]. Recently, an outbreak of chikungunya in Emilia-Romagna, Italy, was reported in 2007 [26]. These cases emphasize the importance of investigating viral infections in febrile travellers, the potential for local outbreak of viral infections, and the necessity of maintaining active surveillance in non-endemic countries.

Surveillance of imported virus infections and control of potential local outbreaks of exotic infections are challenging in many ways. Clinicians might not be aware of the risk and a targeted diagnostic approach might be delayed. To prevent dissemination, there is a pressing need that suspect cases be referred rapidly to selected centers with full diagnostic containment and treatment potential. It is imperative that along with intensified surveillance of outbreaks in humans and other vertebrate hosts, investigations be strengthened to quantify the risk associated with the presence and geographic distribution of vector species. Given that there are no effective vaccines yet against the main vector-borne diseases, prevention relies heavily on vector control and protection of humans from the bites of infected mosquitoes. In this regard, detailed background information on vector distribution, density, and migration dynamics are needed by public health authorities to develop efficient preparedness plans and implement effective emergency actions to be applied at national and international scales.

Unfortunately, the toolbox for vector control is very small. This is because the efficacy of chemical control is weakened due to the emergence of insecticide resistance in mosquitoes, and the increased awareness of the adverse impacts of insecticide use on the environment. There are few insecticides approved for public health use, and the results from several countries with active monitoring programs are indicating already the emergence of insecticide resistance in *Ae. albopictus* [27]. Furthermore, while local/regional efforts are commendable, it is important to plan for area-wide control programs that transcend national boundaries. This is because of the extensive “mobility” associated with both the mosquitoes and the viremic people traveling in and out of regions endemic for disease. While the use of classical control tools are essential at the present time, significant progress is being made in new innovative methods that build on expanding knowledge on vector–pathogen dynamics. These include means to modulate the pathogen transmission ability of insects, microbial control strategies that block pathogen transmission in insects, and the use of genetic methods such as sterile insect technique (SIT) or incompatible insect technique (IIT) to reduce reproductive capacity of mosquitoes [28]–[30]. The strong association of mosquitoes that transmit the dengue and chikungunya viruses with crowded urban environments increases the magnitude of the public health impact of potential outbreak(s). It also poses a greater challenge for the application of vector control methods that utilize classical insecticide-based approaches than those that eliminated malaria in the past. Fighting off this next challenge will require strong collaborations between virologists, entomologists, infectious diseases specialists, veterinary and public health institutions, and media. An interdisciplinary surveillance and control task force working with an informed public may have the best chance of winning the battle against this new threat in Europe.
